# Cerebral Inflammation in an Animal Ischemia–Reperfusion Model Comparing Histidine-Tryptophan-α-Ketoglutarate and Del Nido Cardioplegia

**DOI:** 10.3390/life15030451

**Published:** 2025-03-13

**Authors:** Kristin Klaeske, Maja-Theresa Dieterlen, Jagdip Kang, Zoe Detzer, André Ginther, Susann Ossmann, Michael A. Borger, Philipp Kiefer, Alexandro A. Hoyer

**Affiliations:** Department of Cardiac Surgery, Helios Clinic, Heart Center Leipzig, Leipzig University, 04289 Leipzig, Germany

**Keywords:** Custodiol, del Nido, organ preservation, cardiopulmonary bypass

## Abstract

Brain injury and cerebral inflammation are frequent complications following cardiopulmonary bypass (CPB) resulting in neurocognitive dysfunction, encephalopathy, or stroke. We compared cerebral inflammation induced by del Nido and histidine-tryptophan-α-ketoglutarate (HTK) cardioplegia in a porcine model. Pigs underwent 90 min cardiac arrest using HTK (n = 9) or Jonosteril^®^-based del Nido cardioplegia (n = 9), followed by a 120 min reperfusion. Brain biopsies were collected and analyzed for the mRNA and protein expression of hypoxia-inducible factor-1α (HIF-1α) and cytokines. HTK induced a decrease in blood sodium, chloride, and calcium concentration (cross-clamp aorta: *p*_sodium_ < 0.01, *p*_chloride_ < 0.01, *p*_calcium_ < 0.01; 90 min ischemia: *p*_sodium_ < 0.01, *p*_chloride_ < 0.01, *p*_calcium_ = 0.03) compared to the more stable physiological electrolyte concentrations during del Nido cardioplegia. Hyponatremia and hypochloremia persisted after a 120 min reperfusion in the HTK group (*p*_sodium_ < 0.01, *p*_chloride_ = 0.04). Compared to del Nido, a higher mRNA expression of the proinflammatory cytokine IL-1β was detected in the frontal cortex (HTK: ∆Ct 6.5 ± 1.7; del Nido: ∆Ct 8.8 ± 1.5, *p* = 0.01) and the brain stem (HTK: ∆Ct 5.7 ± 1.5; del Nido: ∆Ct 7.5 ± 1.6, *p* = 0.02) of the HTK group. In conclusion, we showed comparability of HTK and del Nido for cerebral inflammation except for IL-1β expression. Based on our study results, we conclude that del Nido cardioplegia is a suitable and safe alternative to the conventional HTK solution.

## 1. Introduction

Brain injury and cerebral inflammation after cardiac surgery are common complications associated with acute and long-term clinical outcomes and impaired quality of life. While clinically apparent ischemic stroke occurs in 2–5% of patients, neurological dysfunctions such as encephalopathy or neurocognitive dysfunction are more common and occur in up to 32% of patients [1,2].

Brain injury from cardiopulmonary bypass (CPB) is thought to result from microvascular changes, including cerebral blood flow, microembolism, macroembolism, and systemic inflammatory responses. These mechanisms may induce pathophysiological changes in neurological and cognitive impairments during and after cardiac surgery [3,4].

Older age, trauma of surgery, hypertension, and diabetes are postulated risk factors for neurological complications after cardiac surgery [5]. However, reducing the source of injury and increasing the brain tolerance to ischemic insults are the goals of brain protection during cardiac surgery.

The application of a cardioplegic solution extends the time of ischemia tolerance in the heart to protect against myocardial injury during CPB [6]. The inflammatory response to cardiac surgery and CPB makes the blood–brain barrier vulnerable. Therefore, reducing systemic inflammation is vital to minimize cerebral injury [7]. Del Nido cardioplegia was mainly developed in clinical practice for the pediatric population to address the protection of immature cardiomyocytes but is increasingly being used in adult patients [8]. However, conventional histidine-tryptophan-ketoglutarate (HTK) cardioplegia has been widely used, safe, and effective to induce cardiac arrest during open-heart surgery over the last few decades [9].

To improve the knowledge about brain injury following cardiac arrest with different cardioplegic solutions, this study aimed to compare the HTK and del Nido cardioplegia regarding the effects of cardioplegia in adult cardiac surgery on brain injury, particularly on altered oxygen conditions and cerebral inflammation after CPB surgery, in different brain regions during CPB surgery.

## 2. Materials and Methods

### 2.1. Animals and Anesthesia Management

The animal study was performed in accordance with the directive 2010/63/EU on the protection of animals used for scientific purposes. The experiments were authorized by the local animal welfare agency in Leipzig, Germany (vote no. TVV 23/19).

Pigs were overnight fasted for about 15 h. They had ad libitum access to drinking water. Prior to transport, 4–5-month-old, healthy female Landrace pigs (50–60 kg) were sedated (midazolam, 0.5 mg/kg (ratiopharm GmbH, Ulm, Germany); atropine, 0.02 mg/kg (B.Braun, Melsungen, Germany); ketamine, 15 mg/kg i.m. (Serumwerk Bernburg AG, Bernburg, Germany)). Afterwards, propofol (2 mg/kg i.v., Fresenius SE & Co. KGaA, Bad Homburg, Germany) and sufentanil (0.5 µg/kg i.v.) were administered via the lateral auricular vein. The pigs were intubated and mechanically ventilated using a Cato anesthesia ventilator (Draeger, Lübeck, Germany) with 50–60 Vol% oxygen, a respiratory rate between 15 and 30, and a tidal volume of 6–10 mL/kg bodyweight. An 8F venous port was placed in the right external jugular vein for the placement of a 7F Swan-Ganz catheter (Teleflex Inc. Wayne, PA, USA) for the measurements of pulmonary artery pressure.

Further, an 8F three-lumen central venous catheter (7F, 20 cm, Teleflex Inc.) was placed in the right external jugular vein for the administration of medications and the calibration of the PiCCO system (PULSION Medical Systems, Feldkirchen, Germany). Arterial blood pressure, cardiac output (CO), and the systemic vascular resistance index (SVRI) were invasively measured via a 6F PiCCO^®^ Catheter (PULSION Medical Systems) in the right femoral artery. We also placed an 8F arterial port in the carotid artery to place a 7F pressure–volume loop catheter for additional hemodynamic measurements with the Sigma^®^ pressure–volume loop (PV-Loop) system (CD Leycom, Hengelo, The Netherlands). Anesthesia was maintained with propofol (30–35 mg/kg/h i.v.) and sufentanil (0.5–2 µg/kg/h i.v.). During mild hypothermia (34 °C), propofol was reduced to 25 mg/kg/h. Prior to extracorporeal circulation, heparin (300 IU/kg i.v.) was administrated to prevent catheter clotting. A whole electrolyte solution (10–20 mL/kg/h; Serumwerk Bernburg AG, Bernburg, Germany) was used for fluid substitution.

### 2.2. Surgical Technique, Extracorporeal Circulation, and Perfusion

First, the pigs underwent median longitudinal sternotomy and pericardiotomy. For extracorporeal circulation, purse-string sutures (Sorin Group; Milan, Italy and EUROSETS, Medolla, Italy) were placed in the aorta ascendens and the right atrium for arterial cannulation with 25 cm × 22F, FemFlex™ (Edwards Lifesciences, Irvine, CA, USA) and venous cannulation with 29 cm × 29F Trim-Flex Dual Stage Venous Drainage Cannula (Edwards Lifesciences). The hemodynamic parameters of heart rate, blood pressure, CO, SVRI, pulmonary artery pressure, heart index, electrocardiography, body temperature, O_2_ saturation, and respiratory parameters were recorded. Blood gasses, pH, lactate, hemoglobin, and electrolyte concentrations were measured in 0.5 mL blood samples using an ABL90 (Radiometer GmbH, Krefeld, Germany). A 12GA DLP^®^ needle vent (Medtronic, Dublin, Irland) for the infusion of cardioplegia was placed at the ascending aorta.

### 2.3. Study Design

All animals were equilibrated for 40 min before cross-clamping the aorta and infusing the cold, single-dose cardioplegic solution. Before starting the experiment, animals were allocated to two experimental groups (n = 9 per group) receiving either Custodiol^®^/histidine-tryptophan-α-ketoglutarate (HTK group) (Dr. Franz Köhler Chemie GmbH, Bensheim, Germany) or Jonosteril^®^-based del Nido solution (del Nido group) (Fresenius SE & Co. KGaA). The del Nido cardioplegic solution was prepared by mixing four parts of Jonosteril^®^ with one part of fully oxygenated porcine blood (1:4, blood:crystalloid solution) (Table 1).

During CPB, hemofiltration was performed to minimize the risk of volume overload. In this ischemia–reperfusion model, the hearts were arrested for 90 min at 34 °C body temperature. Subsequently, 30 min after reperfusion, the body temperature was restored to physiological conditions. Thereafter, the animals were weaned from CPB within 15 min and recovered for 60 min. After 120 min of total recovery, the pig was euthanized, and biopsies were obtained.

Additional pigs were used to obtain whole blood for transfusion to optimize the experimental conditions and to ensure the comparability and hemodynamic stability of the two experimental groups.

### 2.4. Randomization and Blinding

Simple randomization was performed to secure the generation of an unpredictable allocation of animals for each group. Due to the differences in the preparation of the cardioplegic solutions, the complete blinding of the surgical team was impossible. The experimenters were blinded to the group allocation until statistical analysis.

### 2.5. Porcine Brain Preparation

After euthanasia, the brain was immediately extracted by dissecting the skin and muscles of the cranium and performing a craniotomy to remove the cranial bone above the eyes. The brain was removed from the skull by dissecting the olfactory and optic nerves and the spinal cord. Both hemispheres were separated using a scalpel. The brain region’s frontal cortex, cerebellum, brain stem, diencephalon, and colliculus superior of the right hemisphere were prepared, snap-frozen in liquid nitrogen, and stored at −80 °C until further analysis. The left hemisphere was fixed in 4% formaldehyde/phosphate-buffered saline (PBS) with a pH of 7.4 for 36–48 h. Following fixation, the brain region’s frontal cortex, cerebellum, brain stem, diencephalon, and colliculus superior were dissected before embedding in paraffin.

### 2.6. RNA Isolation and Quantitative Real-Time PCR

RNA was isolated from ≤100 mg of snap-frozen brain tissue followed by cDNA synthesis as described previously [10]. For RT-qPCR, the SYBR^®^ Green PCR Master Mix (Life Technologies, Carlsbad, CA, USA) was utilized to quantify the mRNA levels of hypoxia-induced factor-1α (HIF-1α), tumor-necrosis factor-α (TNF-α), interleukin (IL)-10, IL-1β, and IL-1-receptor antagonist (IL-1RN), according to the manufacturer’s instructions (Table 2). Data were normalized to hypoxanthine-guanine phosphoribosyltransferase-1 (HPRT1) expression. ΔCt values were used to calculate relative expression levels. Primers were purchased from TIB MOLBIOL (Berlin, Germany).

### 2.7. Immunohistochemical Analysis and Evaluation

Paraffin-embedded brain tissue was sectioned (3 µm) and stained, as described previously, with some modifications [11]. Cross-sections were incubated with primary antibody against HIF-1α (1:50, Santa Cruz Biotechnology, Dallas, TX, USA) or TNF-α (1:250, Biolegend, San Diego, CA, USA) overnight at 4 °C. After repeated washing with TBS, the sections were incubated with a secondary IgG-biotin-labeled antibody (1:300, Sigma-Aldrich, St. Louis, MO, USA) and afterwards with horseradish peroxidase-conjugated streptavidin (1:200, Thermo Fisher Scientific, Waltham, MA, USA) for 60 min each. The histological evaluation was performed using the Axio Plan 2 microscope (Carl Zeiss AG, Jena, Germany) AxioVision Release 4.8.2 SP3 (Carl Zeiss AG) and ImageJ 2.0.0 software (US National Institutes of Health, Bethesda, MD, USA).

The immunohistochemical TNF-α staining was analyzed using Adobe Photoshop CS2 software (version 9.0). A standardized color mask was created by manually selecting the optically positively stained areas (red to brown) of an image. The positive areas were marked and quantified as a proportion of the overall image based on the number of pixels. HIF-1α staining was analyzed using the visual counter software. The evaluation comprised counting HIF-1α-positive cell nuclei (red to brown) in relation to the total number of cell nuclei.

### 2.8. Enzyme-Linked Immunosorbent Assay (ELISA)

Snap-frozen brain tissue (≤100 mg) samples were homogenized in lysis relaxing buffer and further processed as previously described [9]. The total protein concentrations were determined using the PierceTM Microplate BCA Protein Assay Kit (Thermo Fisher Scientific Inc.), according to the manufacturer’s instructions.

After the extraction of protein from the brain tissue samples, the cytokines IL-10, IL-1β, and IL-1RN were quantified using ELISA kits. The quantifications were performed by using the IL-10 ELISA Kit (antibodies-online GmbH, Aachen, Germany), the IL-1β ELISA Kit (BIOZOL Diagnostica GmbH, Hamburg, Germany), and the IL-1RN ELISA Kit (antibodies-online). All ELISAs were performed according to the manufacturer’s instructions. Samples were previously diluted 1:10 for IL-10 and IL-1β and 1:20 for IL-1RN. Measurements were recorded at 450 nm with the microplate reader Infinite™ 200 PRO and i-control™ software version 1.12 (Tecan, Männedorf, Switzerland).

### 2.9. Statistical Analysis

Statistical analyses were performed using the SPSS Statistics 28 software (IBM, Armonk, NY, USA). Data are represented as mean ± standard deviation unless stated otherwise. *p* values ≤ 0.05 (two-sided) were considered statistically significant.

Two-group comparisons of means were performed with the unpaired Student’s *t*-test for metric parameters. Pearson’s Chi-squared test, Fisher’s exact test, or the Yates continuity correction were used for categorical data.

## 3. Results

### 3.1. Hemodynamic Parameters

There was no significant difference between the HTK and the del Nido group regarding heart rate (*p* = 0.99), diastolic (*p* = 0.69) and systolic blood pressure (*p* = 0.75), mean arterial pressure (*p* = 0.81), central venous pressure (*p* = 0.58), cardiac output (*p* = 0.36), systemic vascular resistance index (*p* = 0.42), systolic (*p* = 0.74), diastolic (*p* = 0.42), and mean pulmonary artery pressure (*p* = 0.60), as well as heart index (*p* = 0.30) during the equilibration period and after 120 min of reperfusion (Table 3)

In addition to hemodynamic parameters, we investigated the effects of HTK and del Nido cardioplegia on myocardial protection separately [12].

In summary, pressure–volume measurements revealed better systolic and diastolic left ventricular performance in del Nido compared to HTK (both *p* < 0.05). Myocardial injury markers, oxidative and nitrosative stress, mitochondrial membrane integrity, as well as apoptosis markers were comparable between del Nido and HTK (all *p* > 0.05).

**Figure 1:**
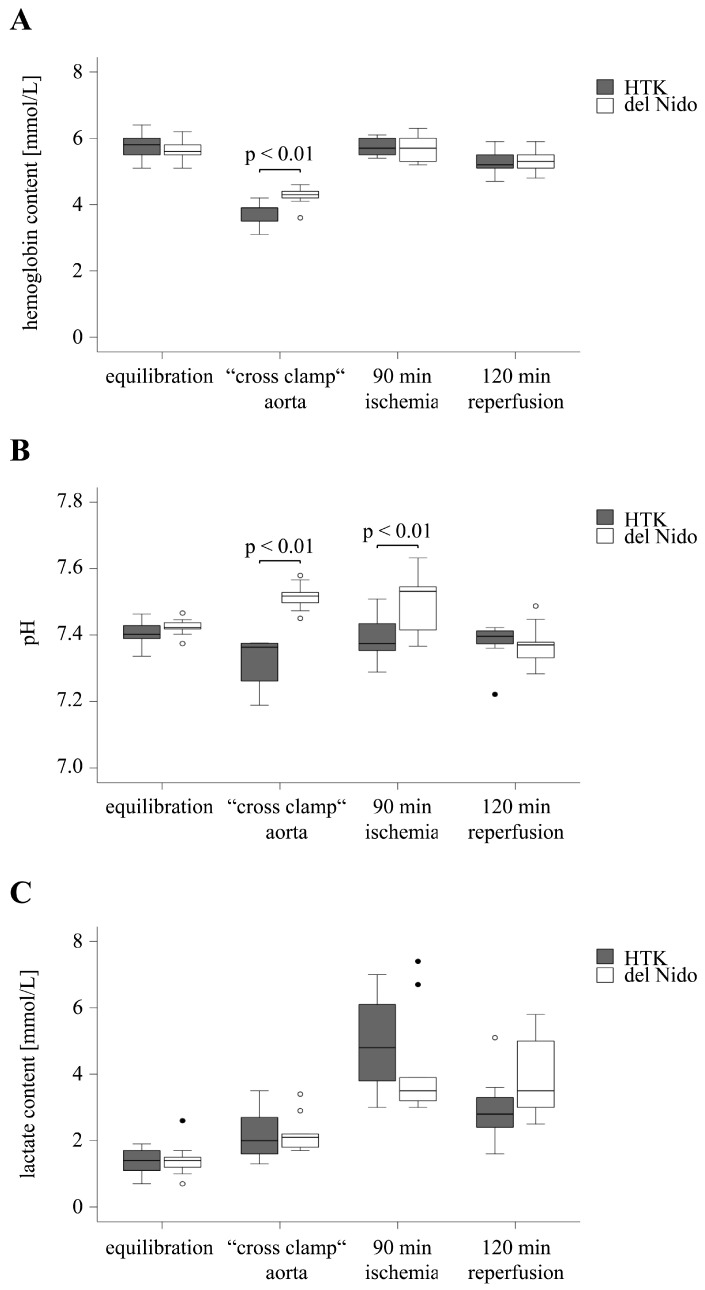
Hemoglobin content (**A**), pH (**B**), and lactate content (**C**) at equilibration, aortic cross-clamping, after 90 min of ischemia, and after 120 min of reperfusion. Dots indicate outliers. HTK, histidine-tryptophan-α-ketoglutarate.

The hemoglobin content was comparable between both groups during equilibration (HTK: 5.8 ± 0.4 mmol/L, del Nido: 5.6 ± 0.3 mmol/L, *p* = 0.43). At the beginning of the ischemic period, a reduction in hemoglobin was observed in both groups, with a significantly lower content in the HTK group compared to the del Nido group (HTK: 3.7 ± 0.4 mmol/L, del Nido: 4.2 ± 0.3 mmol/L, *p* < 0.01). The hemoglobin content returned to baseline level at the end of ischemia (HTK: 5.7 ± 0.3 mmol/L, del Nido: 5.7 ± 0.4 mmol/L, *p* = 0.75) and during the reperfusion period (HTK: 5.3 ± 0.4 mmol/L, del Nido: 5.3 ± 0.3 mmol/L, *p* = 0.90) (Figure 1A).

The pH values of the HTK and del Nido groups were comparable in the equilibration phase (HTK: 7.41 ± 0.04, del Nido: 7.42 ± 0.03, *p* = 0.30) and were within the normal range of 7.39 to 7.45 for arterial blood analysis. During ischemia, the pH differed between both groups (cross-clamp aorta: HTK: 7.31 ± 0.08, del Nido: 7.52 ± 0.04, *p* < 0.01; 90 min ischemia: HTK: 7.39 ± 0.07, del Nido: 7.51 ± 0.09, *p* < 0.01). The pH values stabilized in both groups during the 120 min of reperfusion period (HTK: 7.38 ± 0.06, del Nido: 7.37 ± 0.06, *p* = 0.87) (Figure 1B).

Blood lactate concentrations did not differ between the groups during equilibration (HTK: 1.4 ± 0.4 mmol/L, del Nido: 1.4 ± 0.5 mmol/L, *p* = 0.77), at aorta cross-clamp (HTK: 2.2 ± 0.8 mmol/L, del Nido: 2.2 ± 0.6 mmol/L, *p* = 0.95), after 90 min of ischemia (HTK: 4.9 ± 1.5 mmol/L, del Nido: 4.2 ± 1.7 mmol/L, *p* = 0.32), and after 120 min of reperfusion (HTK: 3.0 ± 1.0 mmol/L, del Nido: 4.0 ± 1.3 mmol/L, *p* = 0.08) (Figure 1C).

In summary, the hemoglobin content and pH values of the HTK group decreased after the application of the cardioplegic solution compared to the del Nido group.

### 3.2. Analysis of the Electrolyte Balance

**Figure 2:**
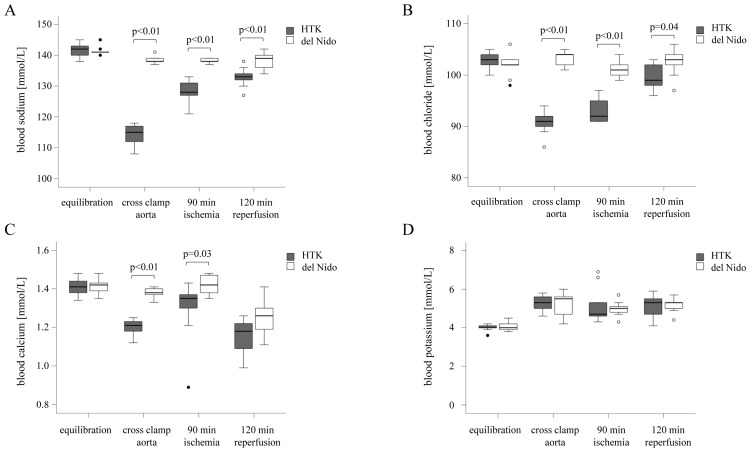
Blood electrolyte concentrations of sodium (**A**), chloride (**B**), calcium (**C**), and potassium (**D**) at equilibration, aortic cross-clamping, after 90 min of ischemia, and after 120 min of reperfusion. Dots indicate outliers. HTK, histidine-tryptophan-α-ketoglutarate.

Blood sodium, chloride, calcium, and potassium levels were comparable between the HTK and del Nido groups during equilibration (*p*_sodium_ = 1, *p*_chloride_ = 0.37, *p*_calcium_ = 0.77, *p*_potassium_ = 0.53). At the beginning of the ischemic period, the levels of sodium and chloride decreased in the HTK group but remained stable in the del Nido group (*p*_sodium_ < 0.01, *p*_chloride_ < 0.01). This difference between the groups persisted during ischemia (*p*_sodium_ < 0.01, *p*_chloride_ < 0.01) until the end of the 120 min of reperfusion period (*p*_sodium_ < 0.01, *p*_chloride_ = 0.04) (Table 4; Figure 2A,B). A significant reduction in calcium concentrations was observed at the beginning of the ischemic period (*p* < 0.01) and during the 90 min of ischemia (*p* = 0.03) (Figure 2C). After 120 min of reperfusion, the calcium levels were comparable in the HTK and del Nido groups (*p* = 0.06). Blood potassium levels were comparable between the groups at all study time points (*p*_equlibriation_ = 0.53, *p*_cross clamp aorta_ = 0.93, *p*_90 min ischemia_ = 0.57, *p*_120 min reperfusion_ = 0.85) (Figure 2D).

In summary, HTK cardioplegia induced a decrease in blood sodium, chloride, and calcium concentration compared to the more stable physiological electrolyte concentrations during del Nido cardioplegia. Hyponatremia and hypochloremia persisted after 120 min of reperfusion in the HTK group.

### 3.3. Analysis of the Hypoxia-Induced Transcription Factor HIF-1α

To assess cerebral hypoxic processes, HIF-1α mRNA expression was analyzed in five different brain regions. HIF-1α expression did not differ between the HTK group (frontal cortex: ∆Ct2.0 ± 1.1, cerebellum: ∆Ct1.4 ± 1.0, brain stem: ∆Ct1.9 ± 1.2, diencephalon: ∆Ct1.9 ± 1.2, and colliculus superior: ∆Ct2.2 ± 1.2) and the del Nido group (frontal cortex: ∆Ct2.5 ± 1.4, *p* = 0.44; cerebellum: ∆Ct1.1 ± 1.4, *p* = 0.58; brain stem: ∆Ct1.2 ± 1.3, *p* = 0.25; diencephalon: ∆Ct1.5 ± 1.5, *p* = 0.55; colliculus superior: ∆Ct1.8 ± 1.8, *p* = 0.63) (Table 5).

**Figure 3:**
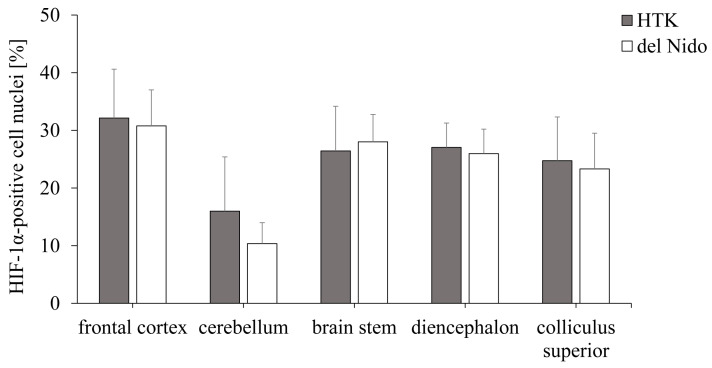
Nuclear HIF-1α translocation in different brain regions after cardioplegia with HTK or del Nido. Proportion of HIF-1α-positive cell nuclei (in %) of the total number of cells. HIF-1α, hypoxia-induced factor-1α; HTK, histidine-tryptophan-α-ketoglutarate.

The translocation of HIF-1α into the nucleus was investigated by immunohistochemistry and is an indicator of hypoxia-induced cellular stress. The HTK and del Nido groups were comparable in terms of nuclear HIF-1α translocation in all brain regions (frontal cortex: *p* = 0.69, cerebellum: *p* = 0.12, brain stem: *p* = 0.59, diencephalon: *p* = 0.58, and colliculus superior: *p* = 0.66) (Figure 3).

In summary, the mRNA and protein expression of HIF-1α did not differ in the HTK and del Nido groups.

### 3.4. Cytokine Expression in the Brain

**Figure 4:**
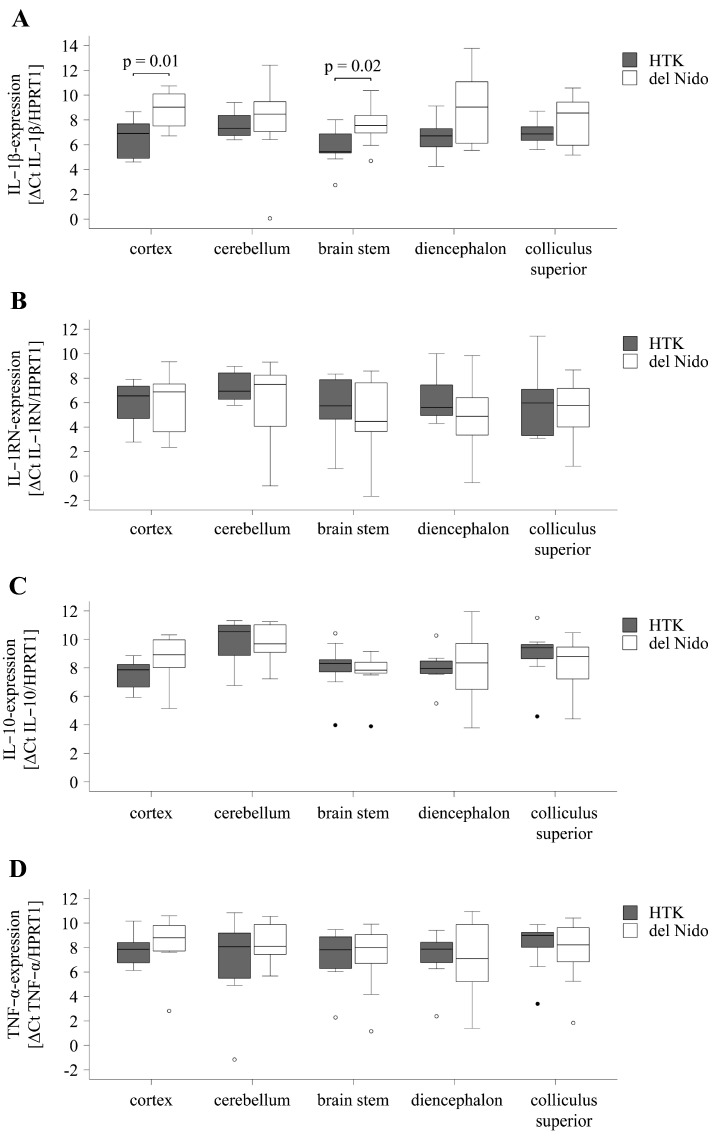
mRNA-expression of IL-1β (**A**), TNF-α (**B**), IL-1RN (**C**), and IL-10 (**D**) in different brain regions after cardioplegia with HTK or del Nido. Dots indicate outliers. ∆Ct, difference in cycle thresholds between the sequence of interest and the reference sequence; HPRT1, hypoxanthine-guanine phosphoribosyltransferase 1; HTK, histidine-tryptophan-α-ketoglutarate; IL-1β/10/1RN, interleukin-1β/10/1RN; TNF-α, tumor necrosis factor-α.

To assess inflammatory processes, the mRNA expression of pro- and anti-inflammatory cytokines IL-1β, TNF-α, IL-1RN, and IL-10 was analyzed in different brain regions. The group comparison revealed comparable expression in the cerebellum (*p* = 0.80), diencephalon (*p* = 0.06), and colliculus superior (*p* = 0.23) and a higher expression of proinflammatory IL-1β in the frontal cortex (HTK: ∆Ct6.5 ± 1.7; del Nido: ∆Ct8.8 ± 1.5, *p* = 0.01) and in the brain stem (HTK: ∆Ct5.7 ± 1.5; del Nido: ∆Ct7.5 ± 1.6, *p* = 0.02) of the HTK group (Figure 4A).

The mRNA expression of the proinflammatory cytokine TNF-α, as well as the anti-inflammatory cytokines IL-1RN and IL-10, was comparable in the different brain regions of both cardioplegia groups (*p* > 0.05) (Figure 4B–D).

The cytoplasmatic expression of the proinflammatory cytokine TNF-α was investigated by immunohistochemistry as a further indicator of cerebral inflammation. The evaluation of TNF-α expression showed that the HTK and del Nido groups were comparable in all brain regions (frontal cortex: *p* = 0.30, cerebellum: *p* = 0.43, brain stem: *p* = 0.95, diencephalon: *p* = 0.45, and colliculus superior: *p* = 0.57) (Table 6).

Further, we found comparability for the protein expression of proinflammatory cytokine IL-1β and anti-inflammatory cytokines IL-1RN and IL-10 between the HTK and del Nido groups (*p* ≥ 0.05) (Table 7).

In summary, the mRNA and protein expression of pro- and anti-inflammatory cytokines TNF-α, IL-1RN, and IL-10 were comparable in different brain regions of the HTK and del Nido groups. Compared to del Nido, a higher mRNA expression of the proinflammatory cytokine IL-1β was detected in the frontal and the brain stem of the HTK group while the protein expression of IL-1β did not differ.

## 4. Discussion

This study compared the effects of the HTK and the del Nido cardioplegic solutions on cerebral inflammation. In our large animal model, a distinct intraoperative electrolyte imbalance was observed in the HTK group. Hemoglobin content and blood pH was more stable with del Nido cardioplegia. HTK and del Nido cardioplegia were comparable regarding hemodynamics, hypoxia-induced cellular processes, and cerebral inflammatory responses except IL-1β.

Hemoglobin concentrations decreased in both study groups at the beginning of the ischemic period, with a greater decrease in the HTK group. The effect of hemodilution during CPB, with a drop of hemoglobin and hematocrit after the administration of crystalloid solution in comparison to blood-based cardioplegic solution, was previously observed [13]. On the one hand, hemodilution improve the microcirculatory flow by reducing blood viscosity and red cell rigidity during hypothermia. On the other hand, a hemodilution-associated anemia may lead to a reduction in the tissue oxygen delivery and postoperative CNS dysfunction [14]. Therefore, mild hemodilution and the maintenance of hemoglobin content, especially at the beginning of cardiac surgery, could help to prevent cerebral inflammation.

Blood pH measurements showed contrary effects after the application of the cardioplegic solution. While the blood pH in the HTK group decreased, which could be explained by the development of metabolic acidosis [15], the blood pH in the del Nido group increased during cardiac arrest. It is well known that the permeability of the blood–brain barrier is pH-dependent, and a reduction in pH can increase the permeability for certain molecules like lactate in the ischemic brain [16]. Currently, the effects of changes in pH on postoperative neurological outcomes are difficult to assess based on the recent literature.

Electrolyte hemostasis in the central nervous system is essential for physiological brain function [17]. An imbalanced electrolyte hemostasis in the periphery will cause imbalances of the electrolyte hemostasis in the brain, leading to direct and indirect effects on neuronal metabolism and function as seen in previous studies [11,17]. In contrast to the physiological electrolyte conditions in the del Nido group, hyponatremia, hypochloremia, and a significant decrease in calcium concentration were observed in the HTK group. Postoperative hyponatremia is associated with the development of cerebral edema because of altered osmolality and can cause brain cell swelling and brain herniation [14,17]. In their report, Crestanello et al. stated that disorders of sodium and osmolality lead to neuronal depression, which correlates with increased mortality, length of hospital stay, and postoperative complications [18].

Kim et al. reported that hyponatremia is associated with postoperative seizures as a result of rapid perioperative changes in sodium levels when HTK is used in pediatric cardiac patients [19].

However, hyponatremia becomes deleterious if osmolality also becomes hypo-osmolar. Lindner et al. showed that the administration of the HTK solution results in isotonic hyponatremia and stable serum osmolality not requiring treatment. In addition, the correction of HTK solution-induced hyponatremia is not recommended, as the rapid normalization of sodium concentration may cause hypertonicity with its associated adverse events [15]. Hypoxia and ischemia interfere with brain adaptation to electrolyte imbalance and exacerbate cerebral edema [17], but we did not detect cerebral edema in any of the investigated brain regions [11]. Hypochloremia is probably explainable by the non-physiological composition of chloride ions in the HTK solution. The decrease in chloride and calcium concentration in the HTK group during ischemia was caused by the calcium- and chloride-reduced HTK solution itself.

The brain is most sensitive to alterations in oxygen delivery and has evolved various adaptive mechanisms to respond rapidly to hypoxia [20]. HIF-1 is a heterodimeric protein complex that is continually synthesized but rapidly degraded under normoxic conditions. In response to low oxygen, a component of HIF, the transcription factor HIF-1α, accumulates in hypoxic cells, translocates into the nucleus, and regulates the activation of a broad range of target genes involved in angiogenesis, cell proliferation, and cell death [21,22]. Rodent studies have demonstrated the induction of the HIF-1α gene and protein expression under hypoxic or ischemic conditions in the brain. Experimental evidence showed increased HIF-1α expression 1 h after systemic hypoxia due to cardiac arrest, elevated levels over 12 h, and persistence for at least 7 d after transient global cerebral ischemia [23]. Therefore, we investigated the HIF-1α nuclear translocation after temporary ischemia induced by cardiac arrest and altered the blood flow conditions in the brain. In our study, HTK- and del Nido-induced cardioplegia resulted in a comparable degree of HIF-1α mRNA expression and nuclear translocation in the analyzed brain regions. It should be noted that the regulation of HIF-1α protein expression under hypoxia occurs primarily post-translationally through the inhibition of proteasomal degradation, whereas transcriptional mechanisms are still under debate [22]. Thus, it can be concluded that the degree of oxidative stress and hypoxic effects induced by cardiac arrest is comparable between HTK- and del Nido-induced cardioplegia.

In addition to hypoxia, the synthesis and activation of HIF-1α can be directly influenced by inflammatory stimuli such as cytokines or growth factors. The stabilization of HIF-1α by IL-1β and TNF-α indicates a central role of this transcription factor in inflammatory processes. The effects of hypoxia during CPB on neuronal tissue are exacerbated by the release of many inflammatory mediators from neuronal cells. Cytokines, such as TNF-α and IL-1β, are known to be released in the early stages of hypoxia, causing either local or systemic inflammation [24].

Cerebral inflammation is a main problem after cardiac surgery and CPB. The inflammatory response includes the activation of coagulation factors as well as the recruitment and adhesion of platelets and leukocytes, which accumulate in cerebral capillaries and thereby impair cerebral blood flow to the injured tissue after CPB [25]. Proinflammatory cytokines such as IL-1β and TNF-α play a central role in the initiation and maintenance of cerebral inflammation. Both cytokines are expressed at constitutively low levels in the healthy brain [26] and are rapidly upregulated after transient cerebral ischemia [11,25,27]. In accordance with the literature, we found different levels of cytokine expression in different brain regions [11,28]. In the present study, there were no significant differences in TNF-α expression between both cardioplegia groups at either mRNA or protein levels in any of the investigated brain regions. However, we documented a significant increase in IL-1β mRNA expression in the frontal cortex and the brain stem of the HTK group. On the one hand, this temporal discrepancy between the transcriptional and translational upregulation of IL-1β may be explained by the short follow-up period. On the other hand, it is known that an increased mRNA expression does not necessarily lead to increased protein expression.

IL-1β and TNF-α are expressed primarily by microglia and activated macrophages in the acute phase and by astrocytes at more chronic phases [26]. Both cytokines are involved in a variety of cellular activities but differ in their structure, the cellular responses they induce, and the pattern of cerebral leukocyte recruitment. While IL-1β preferentially recruits neutrophils and polymorphonuclear cells, TNF-α appears to promote mononuclear cell infiltration [29]. Yang et al. reported that the activation of microglia positively correlates with microglia density, so the cytokine levels of IL-1β and TNF-α may depend on the differential distribution of microglia in the central nervous system. In order to maintain hemostasis, chronic microglia activation plays a critical role in the pathogenesis of neurodegenerative diseases [30]. Wu et al. showed that neurons respond acutely to ischemic injury by increasing the expression of the transcription factor C/EBPβ, which triggers inflammatory brain damage by elevating the expression of TNFα and IL-1β in neurons [31].

The anti-inflammatory cytokine IL-10 has inhibitory effects on a variety of immune cells to decrease inflammation and limit apoptosis. For example, IL-10 inhibits the activation of microglia as well as the production and release of proinflammatory factors. Previous research has shown that a significant reduction in IL-10 levels is associated with the degree of neurological impairment, and IL-10 levels are highly predictive of early neurobehavioral performance after acute stroke [32].

IL-1RN has been identified as a highly selective, competitive antagonist of the IL-1 signaling pathway. The protective effects of systemic IL-1RN in different models of stroke and other forms of brain injury following cerebral ischemia are widely recognized [33]. In the present study, there were no significant differences in anti-inflammatory IL-10 and IL-1RN expression between both cardioplegia groups.

The effect of gender on cerebral inflammation after cardiac surgery is controversial in the current literature. On the one hand, in a retrospective cohort study with adult cardiac surgery patients, female sex was positively correlated with a higher risk of systemic inflammatory response syndrome compared with male sex [34]. On the other hand, Villa et al. reported in an experimental model that female microglia have neuroprotective properties by limiting brain damage caused by acute focal cerebral ischemia [35]. Another study examined neurocognitive decline after cardiac surgery and showed no significant gender differences after the 1-month follow-up period [36]. Furthermore, the mean age of patients who underwent cardiac surgery with del Nido or HTK cardioplegia was 57. In this context, the postmenopausal female gender may be less significant [37].

In summary, the effects on the inflammatory cascade and, more broadly, on the neurological outcome are difficult to assess, as the two cardioplegia only differed in the IL-1β mRNA expression of two brain regions but not in the protein expression. Therefore, the HTK and del Nido cardioplegia can be considered comparable in terms of cerebral inflammatory effects.

### Limitations

This study included healthy animals with a short follow-up period of 120 min. In clinical practice, pre-existing comorbidities are usual in patients who undergo cardiac surgery. Comorbidities could influence study results and the transferability of the results to humans. Conclusions about the long-term outcomes are limited. On the one hand, longer reperfusion times or chronic animal experiments with postoperative follow-up revealed further group differences. On the other hand, various external influences could increase the biological variability of the animals and overlap the direct effects of cardioplegia. Furthermore, given the group-dependent preparations and surgical procedure, not all participants were blinded until the end of the study. However, the experimenters remained blinded until the completion of the laboratory analysis. Another limitation of our study was using surrogate parameters such as nuclear HIF-1α translocation to measure changes in hypoxic conditions in brain tissue. In addition, due to defined measurement time points, the peak levels of the analyzed parameters were not necessarily recorded.

## 5. Conclusions

Our study showed comparability of the HTK and the del Nido cardioplegia against cerebral inflammation regarding hemodynamic parameters and cerebral oxygenation due to HIF-1α expression after CPB surgery. A lower degree of cerebral inflammatory IL-1β expression and more stable electrolyte hemostasis was found with del Nido cardioplegia. Based on our study results, we conclude that del Nido cardioplegia is a suitable and safe alternative to the conventional HTK solution regarding cerebral inflammation.

## Figures and Tables

**Table 1:** Composition of HTK cardioplegia and del Nido cardioplegia.

	HTK Solution	Jonosteril^®^-Based Del Nido Solution *
Na^+^ [mmol/L]	15	142
K^+^ [mmol/L]	9	28
Cl^−^ [mmol/L]	50	129
Mg^2+^ [mmol/L]	4	9
Ca^2+^ [mmol/L]	0.015	1.564
histidine [mmol/L]	198	0
tryptophan [mmol/L]	2	0
mannitol [mmol/L]	30	17
α-ketoglutarate [mmol/L]	1	0
lidocaine [mmol/L]	0	0.26

* concentrations in the crystalloid solution prior to blood addition. HTK, histidine-tryptophan-ketoglutarate; Na^+^, sodium; Ca^2+^, calcium; K^+^, potassium; Mg^2+^, magnesium; Cl^−^, chloride.

**Table 2:** Primer sequences for quantitative real-time PCR analysis.

Target Gene	Forward Primer 5′-3′	Reverse Primer 5′-3′
HIF-1α	cacacagaaatggccttgtgaa	tgttcatagttctccccctgc
HPRT1	ggacttgaatcatgtttgtg	cagatgtttccaaactcaac
IL-10	ggcgctgtcatcaatttctgc	ggctttgtagacacccctct
IL-1β	cgtgcaatgatgactttgtctgt	tcatgcagaacaccacttctct
IL-1RN	gtcctgttgttgcatggtcac	ggtctctttcccaaggggtg
TNF-α	gcccccagaaggaagagttt	gacattggctacaacgtggg

HIF-1α, hypoxia-induced factor-1α; HPRT, hypoxanthine-guanine phosphoribosyltransferase 1; IL-1β/1RN/10, interleukin-1β/1RN/10; TNF-α, tumor-necrosis factor-α.

**Table 3:** Hemodynamic parameters during equilibration and after 120 min of reperfusion in the HTK and del Nido groups.

	Equilibration Period			120 min Reperfusion		
	HTK n = 9	Del Nido n = 9	*p* Value	HTK n = 9	Del Nido n = 9	*p* Value
HR [bpm]	94.9 ± 16.7	94.9 ± 18.6	0.99	123.5 ± 18.2	112.4 ± 14.1	0.18
RR_sys_ [mmHg]	93.3 ± 18.7	97.1 ± 20	0.69	73.9 ± 14.4	79.9 ± 10.7	0.34
RR_dias_ [mmHg]	50.9 ± 11.4	52.6 ± 10.2	0.75	41.1 ± 3.0	44.1 ± 4.5	0.14
MAP [mmHg]	67.6 ± 14.3	69.3 ± 13.9	0.81	54.1 ± 6.7	59.1 ± 5.8	0.12
CVP [mmHg]	12.5 ± 4.0	13.3 ± 0.9	0.58	17.8 ± 1.8	16.1 ± 1.8	0.08
CO [L/min]	6.0 ± 1.1	6.5 ± 1.1	0.36	5.3 ± 1.4	5.4 ± 1.4	0.85
SVRI [dyn*s*cm^−5^*m^2^]	961 ± 258	877 ± 114	0.42	760 ± 281	830 ± 128	0.53
PA_sys_ [mmHg]	29 ± 8.4	30.4 ± 9.2	0.74	40.8 ± 10.6	42.7 ± 13.2	0.75
PA_dias_ [mmHg]	21.1 ± 5.6	23.2 ± 4.8	0.42	26.4 ± 6.8	26.6 ± 7.5	0.96
PA_mean_ [mmHg]	25.1 ± 6.0	26.8 ± 6.6	0.60	33.8 ± 9.1	33.7 ± 9.4	0.99
HI [(L/min)/m^2^]	4.7 ± 0.8	5.1 ± 0.7	0.30	4.1 ± 1.0	4.3 ± 1.2	0.77

bpm, beats per minute; CO, cardiac output; CVP, central venous pressure; HI, heart index; HR, heart rate; HTK, histidine-tryptophan-α-ketoglutarate; MAP, mean arterial pressure; PA_dias/mean/sys_, diastolic/mean/systolic pulmonary artery pressure; RR_dias/sys_, diastolic/systolic blood pressure; SVRI, systemic vascular resistance index.

**Table 4:** Blood electrolyte concentrations in the HTK and del Nido groups.

	HTK (n = 9)	Del Nido (n = 9)	*p*-Value
sodium [mmol/L]			
equilibration	141.4 ± 2.2	141.4 ± 1.4	1.00
aorta cross-clamp	114.3 ± 3.3	138.3 ± 1.2	<0.01
90 min ischemia	128.6 ± 3.7	138.1 ± 0.8	<0.01
120 min reperfusion	133.0 ± 3.2	138.2 ± 2.9	<0.01
chloride [mmol/L]			
equilibration	102.9 ± 1.7	102.0 ± 2.4	0.37
aorta cross-clamp	90.8 ± 2.3	103.1 ± 1.5	<0.01
90 min ischemia	92.9 ± 2.2	101.3 ± 1.9	<0.01
120 min reperfusion	99.7 ± 2.5	102.3 ± 2.6	0.04
calcium [mmol/L]			
equilibration	1.41 ± 0.04	1.42 ± 0.04	0.77
aorta cross-clamp	1.20 ± 0.05	1.38 ± 0.03	0.01
90 min ischemia	1.29 ± 0.16	1.42 ± 0.05	0.03
120 min reperfusion	1.15 ± 0.09	1.24 ± 0.09	0.06
potassium [mmol/L]			
equilibration	4.0 ± 0.2	4.1 ± 0.2	0.53
aorta cross-clamp	5.3 ± 0.4	5.3 ± 0.6	0.93
90 min ischemia	5.2 ± 0.9	5.0 ± 0.4	0.57
120 min reperfusion	5.1 ± 0.6	5.2 ± 0.4	0.85

HTK, histidine-tryptophan-ketoglutarate.

**Table 5:** ∆Ct values of HIF-1α mRNA expression in the HTK and del Nido group.

Brain Region	HTK (n = 9)	Del Nido (n = 9)	*p*-Value
frontal cortex	2.0 ± 1.1	2.5 ± 1.4	0.44
cerebellum	1.4 ± 1.0	1.1 ± 1.4	0.58
brain stem	1.9 ± 1.2	1.2 ± 1.3	0.25
diencephalon	1.9 ± 1.2	1.5 ± 1.5	0.55
colliculus superior	2.2 ± 1.2	1.8 ± 1.8	0.63

Ct, cycle threshold; HIF-1α, hypoxia-induced factor-1α; HTK, histidine-tryptophan-ketoglutarate.

**Table 6:** Immunohistochemical analysis of TNF-α expression. TNF-α-positive stained areas (in ‰) as a proportion of the overall image based on the number of pixels. Data are presented as mean ± standard error.

TNF-α-Positive Area [‰]	HTK (n = 9)	Del Nido (n = 9)	*p*-Value
frontal cortex	1.2 ± 0.4	0.7 ± 0.2	0.30
cerebellum	0.8 ± 0.2	1.1 ± 0.4	0.43
brain stem	1.8 ± 1.2	1.9 ± 0.6	0.95
diencephalon	0.8 ± 0.4	1.4 ± 0.6	0.45
colliculus superior	0.3 ± 0.1	0.5 ± 0.3	0.57

HTK, histidine-tryptophan-ketoglutarate; TNF-α, tumor necrosis factor α.

**Table 7:** Protein expression of IL-1β, IL-1RN, and IL-10. Data are presented as mean ± standard error.

Cytokine	HTK (n = 9)	Del Nido (n = 9)	*p*-Value
IL-1β [pg/mg protein]			
frontal cortex	3.4 ± 0.7	2.1 ± 0.7	0.23
cerebellum	14.4 ± 5.4	17.9 ± 8.7	0.74
brain stem	33.8 ± 14.5	33.0 ± 12.9	0.97
diencephalon	9.6 ± 2.5	13.7 ± 6.3	0.55
colliculus superior	7.6 ± 1.2	18.9 ± 11.8	0.37
IL-1RN [ng/mg protein]			
frontal cortex	1.9 ± 0.3	2.4 ± 0.5	0.33
cerebellum	4.5 ± 1.1	9.3 ± 2.9	0.16
brain stem	13.4 ± 3.4	15.9 ± 3.3	0.61
diencephalon	9.1 ± 2.0	7.3 ± 2.6	0.60
colliculus superior	7.4 ± 2.6	14.6 ± 6.5	0.33
IL-10 [pg/mg protein]			
frontal cortex	9.5 ± 4.0	10.0 ± 3.3	0.91
cerebellum	45.7 ± 11.1	41.8 ± 9.8	0.80
brain stem	81.0 ± 14.1	100.7 ± 17.6	0.40
diencephalon	33.3 ± 6.4	37.8 ± 11.0	0.73
colliculus superior	34.7 ± 7.1	64.6 ± 22.8	0.24

IL-1β/1RN/10, interleukin-1β/1RN/10; HTK, histidine-tryptophan-ketoglutarate.

## Data Availability

The data underlying this article will be shared upon reasonable request to the corresponding author.

## Abbreviations

The following abbreviations are used in this manuscript:

CPB	cardiopulmonary bypass
DEPC	diethylpyrocarbonate
ELISA	Enzyme-linked immunosorbent assay
HIF-1	hypoxia-inducible factor-1α
HPRT-1	hypoxanthine-guanine phosphoribosyltransferase-1
HTK	histidine-tryptophan-α-ketoglutarate
IL	interleukin
IL-1RN	IL-1-receptor antagonist
IRI	ischemia–reperfusion injury
PBS	phosphate-buffered saline
RT-qPCR	quantitative real-time PCR analysis
TBS	tris-buffered saline
TNF-α	tumor-necrosis factor-α
